# Supplementary material for: Shaping Policy on Chronic Diseases through National Policy Dialogs in CHRODIS PLUS
Source: Int J Environ Res Public Health. 2020 Sep 28;17(19):7113. doi: 10.3390/ijerph17197113 (PMC7579029; doi:10.3390/ijerph17197113)

*SUPPLEMENTARY QUESTIONNAIRE (S3) – CHRODIS PLUS Policy Dialogue Feedback Survey*


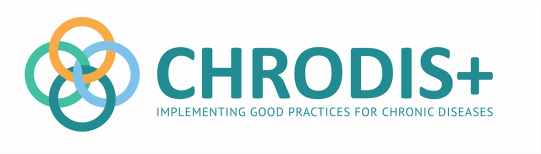
Policy Dialogues Feedback Survey

Thank you for taking the time to provide your valuable feedback relating to the Policy Dialogue you recently attended in the context of CHRODIS Plus Joint Action (2017-2020), implementing good practices for chronic diseases.

To evaluate the effectiveness and relevance of the Policy Dialogues and consultations with experts, we kindly ask for your assistance in completing this feedback survey. Your insights and comments will help shape and strengthen future Policy Dialogues.

The survey should take less than 5 minutes to complete. Your responses to this survey will be kept confidential. Please provide us with the following information about yourself:

| 1. You are: |  | |
| --- | --- | --- |
| Policy maker | MoH technical expert | MoH external advisor |
| MoH minister  Researcher | Minister, other  Other | Healthcare Manager |

2. In which country did you participate?

3. How would you rate each of the following (From 1-Poor to 5-Excellent)

|  | 1 | 2 | 3 | 4 | 5 |
| --- | --- | --- | --- | --- | --- |
| Background information shared in preparation for the Policy Dialogue |  |  |  |  |  |
| Sufficiency of evidence provided and discussed in the Policy Dialogue |  |  |  |  |  |
| Relevance of the topics covered |  |  |  |  |  |

|  | 1 | 2 | 3 | 4 | 5 |
| --- | --- | --- | --- | --- | --- |
| Achievement of goals as delineated in the agenda |  |  |  |  |  |
| Moderation |  |  |  |  |  |
| Definition and agreement of outcomes and action plan to move forward |  |  |  |  |  |
| The location of the Policy Dialogue |  |  |  |  |  |
| Technical conditions (material, etc.) for the Policy Dialogue |  |  |  |  |  |

4. What is, in your opinion, the most relevant topic or idea covered/shared during this Policy Dialogue for you and your country and why?

5. What do you see are the primary enhancing factors and/or barriers for the implementation and sustainability in your National Policies of the outcomes/proposals agreed in this Policy Dialogue?

Enhancing factors:

Barriers:

6. In your opinion, what do you think worked well and what could be improved from this meeting?

7. Overall, how would you rate this Policy Dialogue? (From 1-Very Poor, to 10-Excellent)

1 2 3 4 5 6 7 8 9 10

8. Do you have any further comments or suggestions?

Thank you for taking the time to complete this survey. We truly value the information you have provided. Your responses will contribute to our analysis and help us to improve policy dialogues in the future.


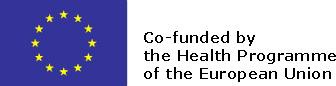

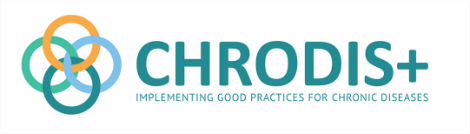

Supplement: Supplementary file 1 [file ijerph-17-07113-s001.zip › ijerph-901791-supplementary/Supplementary Questionnaire_S3_Sienkiewicz.docx]
