# Supplementary material for: Shaping Policy on Chronic Diseases through National Policy Dialogs in CHRODIS PLUS
Source: Int J Environ Res Public Health. 2020 Sep 28;17(19):7113. doi: 10.3390/ijerph17197113 (PMC7579029; doi:10.3390/ijerph17197113)
Supplement: Supplementary file 1 [file ijerph-17-07113-s001.zip › ijerph-901791-supplementary/Supplementary Table 1_S1_Sienkiewicz.docx]

*SUPPLEMENTARY TABLE I (S1) – CHRODIS PLUS Policy Dialogue Topics and Organising Partner*

| Member State | Dialogue Topic | Organising Partner |
| --- | --- | --- |
| Croatia | The implementation of the Action Plan for the Surveillance and Prevention of Chronic non-Communicable diseases 2017-2025 (intersectoral collaboration). | Croatian Public Health Institute |
| Greece | Implementation of Integrated Care Services for the elderly and the chronic diseases patients. | Department of Internal Medicine of Aristotle University of Thessaloniki, AHEPA University Hospital |
| Hungary | How to support the use of artificial intelligence in screening programmes. | Health Services Management Training Centre, Semmelweis University |
| Iceland | How the Health promoting community program (HPCP) can support the implementation of the Sustainable development goals (SDGs), with special focus on SDG no 3. | Directorate of Health, Iceland |
| Ireland | Socio-economic inequalities in tobacco use. | Institute of Public Health, Ireland |
| Italy | Innovation and strategies for chronicity: building alliances to overcome barriers. | National Agency for Regional Healthcare Services and Ministry of Health, Italy |
| Lithuania | Mental health literacy and challenges of going through a divorce – the needs and opportunities for systematic interdisciplinary cooperation. | Lithuanian Institute of Hygiene in association with Faculty of Medicine, Vilnius University |
| Malta | Promoting water consumption and how to improve drinking water infrastructure in public places. | Health Promotion and Disease Prevention, Department of Health |
| The Netherlands | Identifying intersectoral strategies to promote walking and bicycling before, during and after work in The Netherlands to prevent NCDs. | Netherlands National Institute for Public Health and the Environment (RIVM) |
| Poland | Assessing the verification of risk factors and the improvement of the efficiency of health care for the elderly with a high risk of cardiovascular diseases and falls. | National Institute of Geriatrics, Rheumatology and Rehabilitation, Poland |
| Portugal | Advertisement of Food and Beverages to Children. | Directorate of Disease Prevention and Health Promotion, Portuguese Directorate-General of Health |
| Slovakia | Identify the ways to implement a collaborative approach in Control and Prevention of cardiovascular and metabolic disorders. | General Secretary of the Ministry of Health, Slovakia |
| Slovenia | Integrated care in Slovenia. | National institute for Public Health, Slovenia |
| Spain | Health Impact Assessment and alternatives for an effective implementation of Article 35 of the Spanish Public Health Act 33/2011. | Ministry of Health, Consumer Affairs and Social Welfare, |
