# Supplementary material for: Shaping Policy on Chronic Diseases through National Policy Dialogs in CHRODIS PLUS
Source: Int J Environ Res Public Health. 2020 Sep 28;17(19):7113. doi: 10.3390/ijerph17197113 (PMC7579029; doi:10.3390/ijerph17197113)
Supplement: Supplementary file 1 [file ijerph-17-07113-s001.zip › ijerph-901791-supplementary/Supplementary Table 2_S2_Sienkiewicz.docx]

*SUPPLEMENTARY TABLE 2 (S2) – CHRODIS PLUS Policy Dialogue Planning Questionnaire*

| Topic | Question |
| --- | --- |
| 1) Topic / Problem Identification (Context Analysis) | *Which topic will your policy dialogue address? Please explain the policy background.*  *Specify the problem the policy dialogue will address.*  *Please provide any (evidence-based) background material that may be shared with participants.*  *What will be the working title of the dialogue?* |
| 2) Stakeholder Analysis | *Which policymakers do you identify to be relevant? Please give their name, organisation and position.*  *Which stakeholders do you consider relevant for your dialogue? Please give their name, organisation and position.* |
| 3) EU added value | *How will you use EU tools and/or instruments in your policy dialogue?* |
|  | *Do you think your policy dialogue is the appropriate vehicle to provide content for discussion at European level?*  *If so, would you be able to include a slot for a discussion on such content in your policy dialogue?* |
| 5) Logistics  5.1 Language  5.2 External Facilitator  5.3 Timing  5.4 Venue  5.5 Other Needs | *In what language will the policy dialogue be held?*  *Who do you foresee as a facilitator(s)? Please give their name, organisation and position. Who would be a suitable alternative?*  *When do you foresee the policy dialogue to be held? Please note any important events that need to be taken into consideration.*  *Please indicate an alternative date as second option, for overall planning purposes.*  *Where do you plan to hold the policy dialogue? Please state the city and venue/premises.*  *Will you require any other assistance in terms of systems, software, etc? If so, does your organisation or the venue have the necessary equipment?* |
| 6) Draft agenda | *Policy dialogues should last between two and four hours. Please think of a first draft agenda of how you would structure your policy dialogue.* |
| 7) Objectives | *What would your country like to achieve with the policy dialogue? Please define the key objectives.* |
| 8) Outcomes | *What would be your ideal outcomes (in terms of suggested actions)?* |
| 9) Contacts | *Please enter the contact details of the person(s) in charge of the organisation and the content of the Policy Dialogue.*  *Are the contact person(s) above specified willing to collaborate with other project activities providing any further information on the topic discussed during the Policy Dialogue and its findings?* |
